# Supplementary material for: Cryptic Diversity and Demographic Expansion of Plasmodium knowlesi Malaria Vectors in Malaysia
Source: Genes (Basel). 2023 Jun 28;14(7):1369. doi: 10.3390/genes14071369 (PMC10378955; doi:10.3390/genes14071369)
Supplement: Supplementary file 1 [file genes-14-01369-s001.zip › genes-2450523-supplementary/Figure S1.pdf]

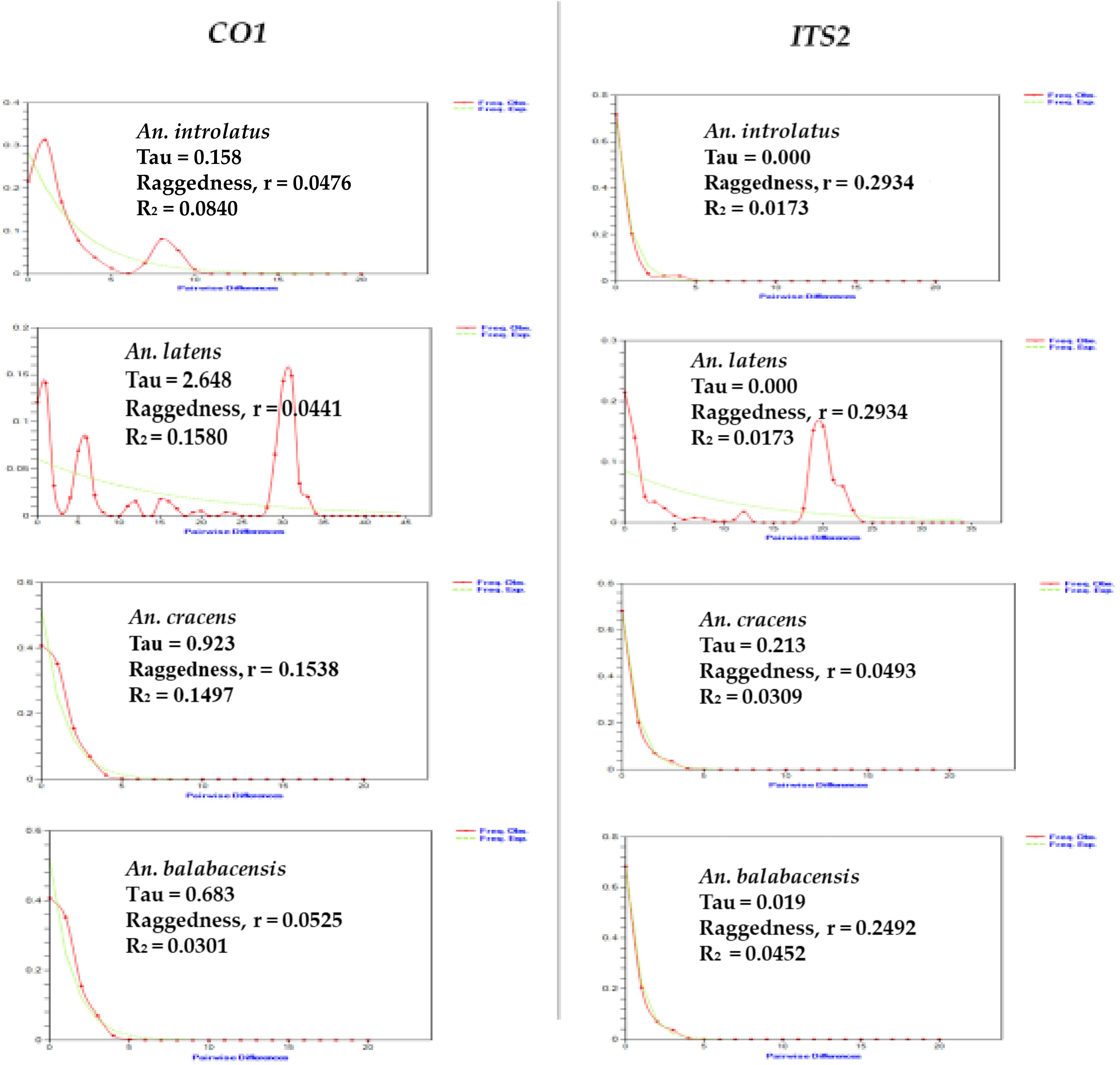

Figure S1. Graphs of the mismatch distribution analysis for (a) *An. introlatus*, (b) *An. latens*, (c) *An. cracens* and (d) *An. balabacensis* based on *COI* and *ITS2*.
