# Supplementary material for: Cryptic Diversity and Demographic Expansion of Plasmodium knowlesi Malaria Vectors in Malaysia
Source: Genes (Basel). 2023 Jun 28;14(7):1369. doi: 10.3390/genes14071369 (PMC10378955; doi:10.3390/genes14071369)
Supplement: Supplementary file 1 [file genes-14-01369-s001.zip › genes-2450523-supplementary/Table S1.pdf]

Table S1. Summary of *CO1* diversity and neutrality test in *An. introlatus*, *An. latens*, *An. cracens* and *An. balabacensis.* Values marked with asterisk indicate significant: *p<0.05, **p<0.01, ***p<0.001.

Species	Subpopulation	No of haplotypes, H	Nucleotide diversity, π	Haplotype diversity, Hd	Neutrality test	
					Tajima's D	Fu's Fs
An. introlatus	**JOHOR**					
	Gunung Panti	2	0.00372 ± 0.00255	0.286 ± 0.196	-1.576	3.644
	Kg. Seri Delima	1	-	-	-	-
	Kem Microwave	9	0.00563 ± 0.00085	0.772 ± 0.052	-0.029	0.605
	Hutan Lenggor	8	0.00264 ± 0.00076	0.648 ± 0.072	-1.674	-2.253
	Kongsi Balak	7	0.00504 ± 0.00102	0.755 ± 0.061	0.503	1.296
	Kg. OA Punjut	1	-	-	-	-
	Kg. OA Berasau	1	-	-	-	-
	Total	11	0.00427 ± 0.00053	0.702 ± 0.039	0.023*	0.082*
	**KELANTAN**					
	Kg. Lalang	2	0.00162 ± 0.00072	0.600 ± 0.215	-1.233	-0.189
	Kg. Dusun Durian	1	-	-	-	-
	Kg. Lebur Jaya	1	-	-	-	-
	Total	3	0.00209 ± 0.00055	0.714 ± 0.123	0.458	0.671
	**PAHANG**					
	Kem Sri Gading	3	0.00256 ± 0.00035	0.689 ± 0.104	1.755	1.4220
	Total	3	0.00256 ± 0.00035	0.689 ± 0.104	1.755	1.4220
	**PERAK**					
	Kg. Sg Dara	1	0.00000 ± 0.00000	0.000 ± 0.000	0.000	0.000
	Kg. Draco	1	0.00000 ± 0.00000	0.000 ± 0.000	-	-
	Total	1	0.00000 ± 0.00000	0.000 ± 0.000	0.000	0.000
	**NEGERI SEMBILAN**					
	Kebun Durian Tekir	1	-	-	-	-
	Hutan Lenggeng	1	0.00000 ± 0.00000	0.000 ± 0.000	0.000	0.000
	Total	1	0.00000 ± 0.00000	0.000 ± 0.000	0.000	0.000
	**SELANGOR**					
	Hulu Kalong	3	0.00195 ± 0.00195	0.800 ± 0.164	-0.065	0.251
	Sg. Sendat	3	0.00352 ± 0.00353	0.833 ± 0.222	1.459	-0.186
	Total	6	0.00559 ± 0.00562	0.917 ± 0.073	0.773*	-0.840*
	**Overall Total**	16	0.00417 ± 0.00420	0.783 ± 0.025	-0.246***	-2.515**
An. latens	**JOHOR**					
	Gunung Panti	4	0.00382 ± 0.00075	0.778 ± 0.091	1.109	2.482
	Total	4	0.00382 ± 0.00075	0.778 ± 0.091	1.109	2.482
	**KELANTAN**					
	Kg. Lalang	4	0.00420 ± 0.00096	0.706 ± 0.075	1.637	1.093
	Total	4	0.00420 ± 0.00096	0.706 ± 0.075	1.637	1.093
	**SABAH**					
	Danum Valley Field Centre	3	0.00351 ± 0.00182	0.524 ± 0.209	-1.576	1.598
	Total	3	0.00351 ± 0.00182	0.524 ± 0.209	-1.576	1.598
	**SARAWAK**					
	Taman Ixora	1	-	-	-	-
	Kg. Sawang	1	-	-	-	-
	Rumah Sewa Panto	4	0.01997 ± 0.00871	1.000 ± 0.177	-0.481	1.598
	Total	5	0.01505 ± 0.00686	0.933 ± 0.122	-1.081	0.784
	**Overall Total**	14	0.02385 ± 0.00279	0.879 ± 0.036	1.237	4.573
An. cracens	**PAHANG**					
	Sg. Ular	2	0.00074 ± 0.00021	0.467 ± 0.132	0.820	0.818
	Kem Sri Gading	3	0.00125 ± 0.00013	0.637 ± 0.038	1.251	1.203
	Total	3	0.00115 ± 0.00011	0.616 ± 0.033	1.097	1.182
	**PERLIS**					
	Perlis	1	0.00000 ± 0.00000	0.000 ± 0.000	0.000	0.000
	Total	1	0.00000 ± 0.00000	0.000 ± 0.000	0.000	0.000

	**Overall Total**	4	0.00146 ± 0.00013	0.702 ± 0.031	0.823	0.738
An. balabacensis	**SARAWAK**					
	Kem Kayu Balak Merarap	4	0.00117 ± 0.00032	0.557 ± 0.092	-0.848	-0.521
	Simpang Utong	3	0.00200 ± 0.00067	1.000 ± 0.272	-	-
	Kebun Ldg Sawit Jelapang	1	0.00000 ± 0.00000	0.000 ± 0.000	-	-
	Total	5	0.00121 ± 0.00025	0.609 ± 0.068	-0.609	-1.344
	**SABAH**					
	Danum valley field centre	5	0.00183 ± 0.00045	0.667 ± 0.113	-0.616	-0.916
	Paradason	4	0.00109 ± 0.00048	0.491 ± 0.175	-1.712	-1.415
	Longgom Besar	1	0.00000 ± 0.00000	0.000 ± 0.000	0.000	0.000
	Tinukadan Laut	2	0.00090 ± 0.00026	0.600 ± 0.175	1.225	0.626
	Mambatu Laut	3	0.00150 ± 0.00041	0.800 ± 0.164	0.243	-0.475
	Narandang	2	0.00150 ± 0.00079	0.500 ± 0.265	-0.800	1.099
	Tomohon	3	0.00120 ± 0.00045	0.700 ± 0.218	-0.973	-0.829
	Minikodong	2	0.00100 ± 0.00047	0.667 ± 0.314	-	-
	Timbang Dayang	2	0.00171 ± 0.00028	0.571 ± 0.094	1.794	2.216
	Limbuak Laut	2	0.00037 ± 0.00027	0.250 ± 0.180	-1.055	-0.182
	Sorinsim	1	0.00000 ± 0.00000	0.000 ± 0.000	-	-
	Sinangip	2	0.00075 ± 0.00040	0.500 ± 0.265	-0.612	0.172
	Lipasu Lama	2	0.00200 ± 0.00094	0.667 ±0.314	-	-
	Paus	2	0.00075 ± 0.00040	0.500 ± 0.265	-0.612	0.172
	Keritan Ulu	1	0.00141 ± 0.00018	0.593 ± 0.050	0.0000	0.000
	Total	10	0.00128 ± 0.00018	0.555 ± 0.056	-1.839*	-7.878**
	**Overall total**	16	0.00141 ± 0.00018	0.593 ± 0.050	-1.968*	-12.546**
