# Supplementary material for: Cryptic Diversity and Demographic Expansion of Plasmodium knowlesi Malaria Vectors in Malaysia
Source: Genes (Basel). 2023 Jun 28;14(7):1369. doi: 10.3390/genes14071369 (PMC10378955; doi:10.3390/genes14071369)
Supplement: Supplementary file 1 [file genes-14-01369-s001.zip › genes-2450523-supplementary/Table S2.pdf]

Table S2. Summary of *ITS2* diversity and neutrality test in *An. introlatus*, *An. latens*, *An. cracens* and *An. balabacensis*. Values marked with asterisk indicate significant: *p<0.05, **p<0.01, ***p<0.001.

Species	Subpopulation	No of haplotypes, H	Nucleotide diversity, π	Haplotype diversity, Hd	Neutrality test	
					Tajima's D	Fu's Fs
An. introlatus	**JOHOR**					
	Gunung Panti	3	0.00073 ± 0.00033	0.524 ± 0.209	-1.237	-0.922
	Kg. Seri Delima	1	-	-	-	-
	Kem Microwave	3	0.00017 ± 0.00017	0.132 ± 0.068	-1.304	-2.149
	Hutan Lenggor	2	0.00006 ± 0.00006	0.045 ± 0.043	-1.115	-1.530
	Kongsi Balak	7	0.00091 ± 0.00091	0.484 ± 0.100	-1.664	-3.802
	Kg. OA Punjut	1	-	-	-	-
	Kg. OA Berasau	1	-	-	-	-
	Total	10	0.00036 ± 0.00036	0.226 ± 0.049	-2.078*	-11.886
	**KELANTAN**					
	Kg. Lalang	1	0.00000 ± 0.00000	0.000 ± 0.000	0.000	0.000
	Kg. Dusun Durian	1	-	-	-	-
	Kg. Lebur Jaya	1	-	-	-	-
	Total	1	0.00000 ± 0.00000	0.000 ± 0.000	0.000	0.000
	**PAHANG**					
	Kem Sri Gading	2	0.00026 ± 0.00026	0.200 ± 0.154	-1.112	0.339
	Total	2	0.00026 ± 0.00026	0.200 ± 0.154	-1.112	0.339
	**PERAK**					
	Kg. Sg Dara	1	0.00000 ± 0.00000	0.000 ± 0.000	0.000	0.000
	Kg. Draco	1	0.00000 ± 0.00000	0.000 ± 0.000	-	-
	Total	1	0.00000 ± 0.00000	0.000 ± 0.000	0.000	0.000
	**NEGERI SEMBILAN**					
	Kebun Durian Tekir	1	0.00000 ± 0.00000	0.000 ± 0.000	-	-
	Hutan Lenggeng	1	0.00051 ± 0.00030	0.400 ± 0.237	-0.817	0.090
	Total	2	0.00037 ± 0.00037	0.286 ± 0.196	-1.006	-0.095
	**SELANGOR**					
	Hulu Kalong	5	0.00513 ± 0.00124	1.000 ± 0.126	-1.193	-1.716
	Sg. Sendat	5	0.00411 ± .00412	1.000 ± 0.126	1.174	-2.116
	Total	10	0.00456 ± 0.00458	1.000 ± 0.045	-1.899*	-7.454*
	**Overall Total**	21	0.00057 ± 0.00057	0.283 ± 0.045	-2.604***	-32.340**
An. latens	**JOHOR**					
	Gunung Panti	2	0.00080 ± 0.00037	0.324 ± 0.136	-0.697	-0.623
	Total	2	0.00080 ± 0.00037	0.324 ± 0.136	-0.697	-0.623
	**KELANTAN**					
	Kg. Lalang	2	0.00100 ± 0.00018	0.533 ± 0.095	1.303	1.029
	Total	2	0.00100 ± 0.00018	0.533 ± 0.095	1.303	1.029
	**SABAH**					
	Danum Valley Field Centre	12	0.00582 ± 0.00099	0.956 ± 0.033	-1.827*	-6.395
	Total	12	0.00582 ± 0.00099	0.956 ± 0.033	-1.827*	-6.395
	**SARAWAK**					
	Taman Ixora	1	-	-	-	-
	Kg. Sawang	1	-	-	-	-
	Rumah Sewa Panto	2	0.00674 ± 0.00358	0.500 ± 0.265	-0.817	3.251
	Total	2	0.00450 ± 0.00290	0.333 ± 0.215	-1.390	3.361
	**Overall Total**	16	0.02072 ± 0.00098	0.785 ± 0.054	0.883	1.901
An. cracens	**PAHANG**					
	Sg. Ular	10	0.00201 ± 0.00051	0.737 ± 0.111	-2.345**	-6.501**
	Kem Sri Gading	3	0.00066 ± 0.00012	0.444 ± 0.071	0.018	0.128
	Total	12	0.00117 ± 0.00023	0.562 ± 0.071	-2.292	-9.187
	**PERLIS**					

	Perlis	4	0.00517 ± 0.00105	0.900 ± 0.161	0.913	0.051
	Total	4	0.00517 ± 0.00105	0.900 ± 0.161	0.913	0.051
	**Overall Total**	15	0.00165 ± 0.00035	0.611 ± 0.067	-2.372**	-10.779**
An. balabacensis	**SARAWAK**					
	Kem Kayu Balak Merarap	1	0.0000 ± 0.0000	0.000 ± 0.000	0.000	0.000
	Simpang Utong	2	0.00127 ± 0.00060	0.667 ± 0.314	-	-
	Kebun Ldg Sawit Jelapang	1	0.00000 ± 0.00000	0.000 ± 0.000	-	-
	Total	2	0.00015 ± 0.00013	0.077 ± 0.070	-1.156	-1.094
	**SABAH**					
	Paradason	1	0.00000 ± 0.00000	0.000 ± 0.000	0.000	0.000
	Limbuak Laut	9	0.00395 ± 0.00080	0.939 ± 0.058	-1.790*	-5.622*
	Kudat	1	0.00000 ± 0.00000	0.000 ± 0.000	0.000	0.000
	Total	9	0.00152 ± 0.00046	0.488 ± 0.109	-2.244**	-6.395**
	**Overall total**	10	0.00091 ± 0.00028	0.317 ± 0.080	-2.351**	-9.865**
